# Nocturnal but not diurnal threats shape stopover strategy in a migrating songbird

**DOI:** 10.1111/1365-2656.70059

**Published:** 2025-05-23

**Authors:** Giuseppe Bianco, Sara Raj Pant, Xiaojia Wu, Susanne Åkesson

**Affiliations:** ^1^ Department of Biology Lund University Lund Sweden; ^2^ Department of Ecology University of Innsbruck Innsbruck Austria

**Keywords:** fuelling, landscape of fear, migration, prey–predator interactions

## Abstract

1. Songbird migration involves frequent migratory flights interrupted by several days of stopover to refuel. For first‐year migratory birds, this entails stopping in unfamiliar locations to exploit local resources and maximise fuelling rates. However, stopovers also pose mortality risks due to predator presence.
2. We aimed to determine whether auditory cues from avian predators with differing hunting strategies elicit distinct anti‐predator responses in European robins (*Erithacus rubecula*) during autumn stopover.
3. We exposed captive first‐year European robins to calls of either a diurnal predator, the Eurasian sparrowhawk (*Accipiter nisus*), which captures prey in flight, or a nocturnal predator, the tawny owl (*Strix aluco*), which relies on pouncing attacks. We monitored changes in daily food intake, body condition, activity levels, and timing of nocturnal activity.
4. Robins react to the perceived risk of predation by the nocturnal predator but do not alter their strategy in response to diurnal predator cues. Specifically, exposure to tawny owl calls led to reduced night‐time activity, lower food intake, and slower fuel accumulation, resulting in poorer body condition by the end of the experiment. Lower body condition after stopover can result in a slower migration pace and consequently later arrival to wintering areas, potentially affecting individual fitness.
5. This novel study highlights the flexibility of avian migration programs in adapting to perceived predation risks based on predator activity time and hunting modalities, and how these adaptations differentially shape stopover strategies.

## Graphical abstract

This novel study reveals that nocturnal, but not diurnal, predation risk shapes stopover strategies in a migratory songbird. By distinguishing predator hunting strategies, European robins differentially adjust fueling and activity patterns to mitigate threats. These findings highlight the flexibility of the avian migration programme and its adaptation to complex predation landscapes.

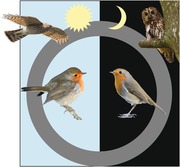

## 1 INTRODUCTION

The ‘landscape of fear’ is a concept in ecology that describes how the presence of predators influences the behaviour and distribution of prey species (Brown et al., 1999; Clinchy et al., 2013; Ripple & Beschta, 2004; Zanette & Clinchy, 2019). This concept extends beyond the direct threat of predation to include the psychological impacts predators have on their prey, profoundly shaping the prey's behaviour and habitat use (Brown et al., 1999). Prey species often allocate their activities in space and time to minimize the risk of predation (Palmer et al., 2022), leading to significant modifications in their daily routines and spatial distribution (Laursen et al., 2016). Fear‐induced changes in prey behaviour can manifest itself in various ways. Foraging behaviour is typically adjusted, with prey often reducing their feeding activities in areas perceived as high‐risk (Gaynor et al., 2019). Habitat use may also shift, as prey avoid regions where predators are frequently encountered (Brown et al., 1999). Increased vigilance is another common response (Brown, 1999), with prey devoting more time to scanning for potential threats, even at the cost of other essential activities (Preisser et al., 2005).

Responses to predation risk arise from a complex interplay of direct and indirect cues, sensory modalities, cue sources and environmental factors (Jones et al., 2024). Anti‐predator responses can carry inherited costs themselves if they fail to provide effective protection against predation (Creel et al., 2019). In some cases, adjustments to predator presence may offer little to no advantage in avoiding predation (Ydenberg et al., 2022) resulting in no real benefit to reduce mortality level (Creel et al., 2019).

Every year, billions of passerine birds migrate thousands of kilometres from their breeding grounds at high latitudes to their wintering grounds in southern territories (Hahn et al., 2009). These massive migrations are timed with the seasonal variation of resource availability and require specialized physiological and behavioural adaptations to timely pursue and adapt to a changing environment while crossing heterogeneous landscapes (Åkesson et al., 2017; Åkesson & Hedenström, 2007; Berthold, 1996; Newton, 2010). Migration is energetically demanding for birds and requires 1 week of foraging at stopover sites to accumulate enough fuel to perform a single day of flight (Hedenström & Alerstam, 1997). Hence, the choice of stopover sites and the strategy to exploit local resources are of utmost importance for migration performances and survival (Alerstam, 2011; Alerstam & Lindström, 1990; Weber et al., 1998). Predation represents the maximum cost on individual fitness. Therefore, it is fundamental for individuals to cope with the presence of predators and adapt, behaviourally and physiologically, to reduce the risk of predation at stopover sites (Alerstam & Lindström, 1990; Lind, 2006; Lind & Cresswell, 2005; Ydenberg et al., 2004). Ultimately, the strategy that birds use to minimize predation risk could potentially affect their overall migration speed (Bayly, 2006; Lindström, 2003; Ydenberg & Hope, 2019) and could have implications for individual fitness (Abbey‐Lee et al., 2016; Zanette et al., 2024).

During migration, some birds can double their body mass by expanding their fat reserves, that is, their primary source of energy for migratory flights (Alerstam, 2011; Bairlein, 2002; Jenni & Jenni‐Eiermann, 1998). Such extreme fuelling drastically increases wing loading, which may compromise flight ability (Hedenström, 1992; Pennycuick, 1989). Heavier birds will suffer from reduced take‐off ability to escape surprise attacks, as demonstrated experimentally by exposing lean and fat birds to simulated predator attacks (Kullberg et al., 1996, 2000; Lind et al., 1999). However, it is also possible that heavy birds compensate behaviourally or physiologically to cope with the risk of predation (Cresswell, 2008; Lind & Cresswell, 2005; Walters et al., 2017). Furthermore, some studies suggest that under the risk of predation, it could be advantageous for lean birds to increase foraging (Metcalfe & Furness, 1984; Moore & Simms, 1986) and attempt to leave the stopover site earlier, even with a lower than optimal fuel load, to find a safer area (Fransson & Weber, 1997). How a first‐year migrant, visiting a stopover site for the first time in its life, can decide on predation risk is still largely unknown. Decisions about foraging strategy, the amount of fuel load and the timing of departure from a stopover site depend on many factors (Alerstam & Lindström, 1990; Ferretti et al., 2019; Schmaljohann & Eikenaar, 2017), but predation risk remains understudied (Alerstam, 2009; Lind et al., 2010).

Auditory cues play a vital role in the predator detection process as many avian predators announce their presence through calls or other sounds (e.g. Zanette et al., 2024). By interpreting these cues, migratory birds can alter their behaviour to reduce the likelihood of encountering predators. However, anti‐predatory responses must be effective in reducing mortality and adapted to specific environments and predator types (Jones et al., 2024). We tested the nocturnally migrating European robin (*Erithacus rubecula*) in a ‘landscape of fear’ scenario (Gaynor et al., 2019) after capture at a stopover location in southern Sweden. We performed two experiments on separate groups of individuals, during the same autumn migratory season. The two predators differ in their time of the day activity and prey‐capture modalities. During the first experiment, we used playback calls of the diurnal avian predator Eurasian sparrowhawk (*Accipiter nisus*), which hunts by pursuing and catching prey in flight. In the second experiment, we presented playback calls of a nocturnal avian predator, the tawny owl (*Strix aluco*) which captures perching birds using a pouncing strategy, striking from above with swift controlled dives. In both experiments, a control group of robins was exposed to playbacks of non‐predatory animals commonly heard in the Swedish forests around the experimental site: call of the common crossbill (*Loxia curvirostra*; Experiment 1) and snorts of a deer (*Cervus* sp.; Experiment 2).

We hypothesize in Experiment 1 that exposure to a diurnal predator will not affect the fuelling strategy of robins, with no differences in food intake and body mass levels from those of the control group. Reducing body mass would not necessarily improve escape manoeuvrability from a pursuing predator like the sparrowhawk; it could instead negatively impact migration speed by increasing the need for additional stopovers. Moreover, we do not expect behavioural changes, predicting that birds in the experimental group will be as active as those in the control group. Any change in migratory restlessness or timing of migration would likely be maladaptive, as robins migrate at night and incur little risk of attacks from a diurnal predator during migratory flights.

In Experiment 2, we hypothesize that exposure to nocturnal avian predator auditory cues will impact food intake and body mass, with experimental birds foraging less and consequently maintaining a leaner body mass compared to control birds. To escape tawny owl pouncing attacks, birds in the experimental group may rely on rapid take‐offs, which could be facilitated by lower body mass. We also predict that birds exposed to nocturnal predators will adjust their restlessness patterns in an attempt to depart earlier and/or prolong nocturnal activity. These behavioural adjustments are expected to minimize encounters with nocturnal predators by ensuring take‐off and landing occur during daylight.

We present our findings in the context of songbirds' stopover ecology, discussing how migratory performance could be influenced by predator‐induced fear. We explore how two different predator‐avoidance strategies may have evolved to cope with the presence of avian predators active at different times of the day. By examining the specific responses to diurnal and nocturnal predators, we provide insights into the adaptive behaviours that enable nocturnal songbird migrants to navigate the complex landscape of fear, optimize their stopover strategies and enhance their overall migration success.

## 2 MATERIALS AND METHODS

### 2.1 Study species and experimental site

European robins (*Erithacus rubecula*) were captured using mist‐nets near Stensoffa Ecological Field Station (55°41′ N, 13°26′ E) in southwestern Sweden during late September and early October 2021. This period coincides with their migration through the area, where they typically spend time refuelling at stopovers. After capture, the birds were aged, ringed and their wing length measured to the nearest millimetre. They were then placed indoors in individual cages with food (mealworms, *Tenebrio molitor*) and water provided ad libitum. Only juvenile robins were used in both experiments, as they are presumed to be naïve to predatory pressure at stopover locations during their first migration. These birds have never visited the experimental stopover before and, therefore, lack any prior knowledge or expectation about predator threats. All birds were moved to the experimental facility on the day the experiments started.

### 2.2 Experimental design

We used two separate groups of 24 individuals, each captured a few days before the start of the relative experiment. At the beginning of each experiment, the 24 individuals were randomly divided into six groups of four robins each. Each group was placed in an independent experimental house located outdoors, several meters away from the nearest building. All six experimental houses were identical in construction (15 m^2^ internal surface, 3.9 m maximum height), featuring a semi‐transparent roof that allowed natural light, and wooden walls with insulating material for acoustic isolation. Inside each house, birds were kept in four individual circular cages (550 mm diameter, 700 mm height), preventing direct visual contact between them or when an operator was accessing the houses (Ilieva et al., 2016, 2018). The birds remained in the experimental houses for 15 days: 1 day for acclimatization and 14 days for data collection. Within 2 days after the experiment ended, all birds were released back into the wild.

In Experiment 1, 12 birds (3 houses) were exposed to recorded playback calls of a diurnal avian predator, the Eurasian sparrowhawk, while 12 control birds (3 houses) were exposed to playback calls of the common crossbill, a seed eater found in similar habitats as robins and present in the forest around the experimental area. In Experiment 2, 12 birds were exposed to playback calls of a nocturnal avian predator, the tawny owl, while another 12 control birds were exposed to the snorting of a deer, a ruminant ungulate of the family *Cervidae*.

**FIGURE 1:**
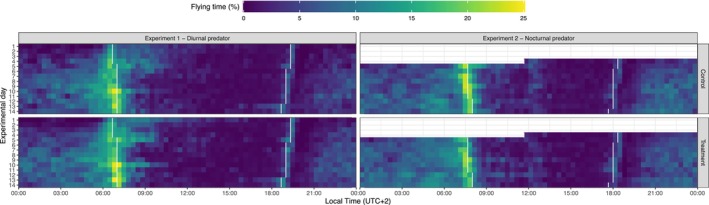
Actogram of first‐year migratory European robins (*Erithacus rubecula*) monitored in two experiments on separate groups of individuals under a playback call of a non‐predator animal (control) and a playback call of either a diurnal or nocturnal avian predator species (treatment). Each horizontal line in each panel shows the mean time spent in flying mode by 12 birds during 24 h in discrete 20‐min intervals. White vertical lines indicate the end of the 20‐min interval corresponding to the local sunrise and the end of the 20‐min interval corresponding to the local sunset. In Experiment 2, the first 3.5 days are missing due to a technical problem with the video‐recording equipment.

Playback calls were played using a Fusion digital playback device (FOXPRO Inc., USA). Calls lasted for 30 s and were repeated every 10 min (totalling 3 min per hour). This short exposure was chosen to avoid inducing chronic stress in the birds. For the diurnal predator experiment, calls were played from local sunrise to sunset (i.e. 12 h per day in average), while for the nocturnal predator experiment, calls were played from sunset to sunrise (13 h per day; see Figure 1). Bird calls were sourced from the CD collection ‘Bird Sounds of Europe and North‐west Africa’ by J.C. Roche and J. Chevereau, while the deer snorting sound was preloaded on the playback device. Audio files were edited using Audacity version 2.4.2 (www.audacityteam.org) to normalize playback volume.

### 2.3 Data collection

Every day at 12:00 (local time, UTC+2), an operator provided fresh food and water, measured daily food intake, and recorded bird weight using an electronic balance beneath each cage. The perch on which birds sat was mechanically anchored to the balance, allowing weight measurement without visual contact with the operator (see Ilieva et al. (2018) for more details). Body condition of individual birds was tracked by normalizing daily body mass by wing length to account for body mass variation due to size differences (Redfem et al., 2000). Body mass at the end of the experiment was also used to estimate potential flight ranges (see below).

We extracted the detailed activity of the four birds in each house using the computer vision procedure presented in Ilieva et al. (2018) applied on the video recorded by the camera installed under each house roof. This computer vision procedure depicts the fractions of time when the bird is in the air, that is, when it is flying from the perch to the edge of the cage, or from one edge of the cage to the perch or to another edge of the cage. This criterion excludes from the activity computation any fluttering and jumping (from and to the perch) and represents a conservative estimate for birds' activity (Ilieva et al., 2018). Raw activity data are plotted in Appendix S1. We used an average of 20‐min intervals (72 intervals per day) to generate the combined actogram in Figure 1 and to perform the calculations and statistical analysis presented below. Due to a technical problem with the video‐recording equipment, the first 3.5 days of activity are missing in Experiment 2 (Figure 1).

Daily activity for each bird was calculated as the mean of all 72 intervals, with separate calculations made for daytime and nighttime periods. We used the 10th percentile of the cumulative nighttime activity to estimate the daily migratory departure time (Schmaljohann et al., 2015) relative to the local sunset. Similarly, we used the 90th percentile of the cumulative nighttime activity to define the end of night activity and calculate the duration of nocturnal restlessness.

### 2.4 Statistical analysis

For all analysis, we used the software R version 4.3.2 (R Core Team, 2023) and the packages detailed as follows. We implemented a series of linear mixed‐effects models using the *lme4* package version 1.1‐35.3 (Bates et al., 2015). We compared models containing relevant predictors and random effects against null models, which included only the relevant random effects, using the likelihood‐ratio test (Tredennick et al., 2021). The models for testing our hypotheses featured one of the following continuous response variables: food intake, body condition, activity level, departure time, duration of nocturnal restlessness. The fixed predictors included in all models were experimental group (treatment or control; categorical variable), experimental day (continuous variable) and the additive interaction between them. In all models, we accounted for repeated measures by including a random intercept for bird ID (categorical) and controlled for pseudoreplication by adding an additional random intercept for experimental house (categorical). In models with body condition as the response variable, food intake (continuous) and an additional additive interaction between group and food intake were included as fixed predictors (see also Huffeldt et al., 2024); we accounted for repeated measures and differing initial masses of each bird by including a random intercept for bird ID (categorical) and a random slope for day (continuous).

We assessed the assumptions of the linear mixed‐effects models by examining histograms of residuals using the *performance* package version 0.11.0 (Lüdecke et al., 2021). When models containing fixed effects explained more variance than the null model, we calculated the marginal means of model predictions and their 95% CIs with the *emmeans* package version 1.10.1 (Lenth, 2023) and plotted them with the *ggplot2* package version 3.5.1 (Wickham, 2016). Predictors were deemed significant at *α* = 0.05 when model coefficients (*β*) 95% confidence intervals (CI) did not encompass zero. Model summary statistics and significance levels were obtained with the *report* package version 0.5.8 (Makowski et al., 2023). Raw data and the R script for building, inspecting, testing, predictors calculations and plots are archived online (Bianco et al., 2025). Flight ranges were computed after Pennycuick (2008) with the package *FlyingR* version 0.2.2.

### 2.5 Ethics statement

Permissions were given by the Malmö/Lund Ethical Committee for scientific work on animals (Dnr 5.8.18‐12719/2017; 5.8.18‐09591/2021), the Swedish Board of Agriculture for housing facilities (Dnr 5.2.18‐5398/16; 5.2.18‐04121/2019) and work with animals (Dnr 5.2.18‐10992/18), the Swedish Nature Protection Agency and the Swedish Ringing Centre (No. 440) for catching and ringing birds.

## 3 RESULTS

Robins exhibited a typical nocturnal migratory pattern in the combined actogram (Figure 1). In both experiments and across treatment groups, birds initiated their migratory activity around sunset. After a quiescent period of 20–30 min, they became actively migratory (i.e. displaying restlessness), with their activity intensifying throughout the night and peaking around local sunrise (Figure 1). At sunrise, robins are expected to land, and the morning activity recorded in the actogram likely corresponds to their settling period, during which they search for a suitable stopover area and engage in foraging activities (Figure 1). The activity level was minimal during the afternoon, indicating this time as their preferred resting period (Figure 1).

**FIGURE 2:**
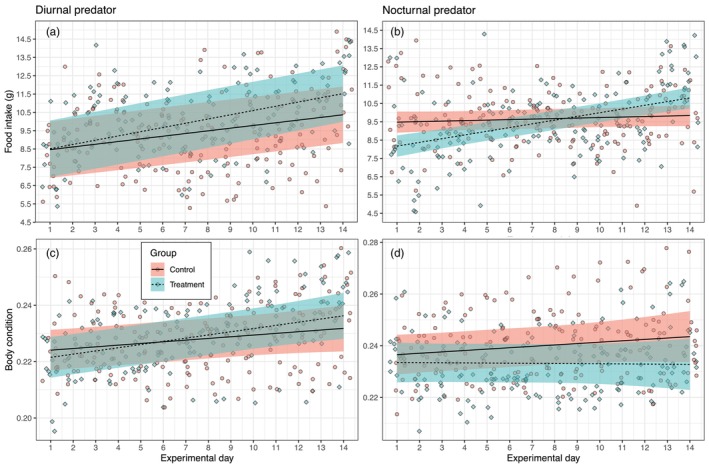
Changes in food intake and body condition (size‐corrected body mass) of first‐year migratory European robins (*Erithacus rubecula*) monitored in two experiments on separate groups of individuals under the playback calls of a non‐predator animal (control) and either a diurnal or nocturnal avian predator species (treatment). Black lines represent linear mixed‐effects model predictions, and shaded areas indicate the 95% confidence intervals of models that explained significant variation in the response variables compared to a null model. Individual raw data points are also displayed in the background. Models' construction, likelihood‐ratio test and significant model estimates are reported in the text.

In models of food intake, the experimental group and the interaction between the experimental group and day explained significant variation in the response variable; models including these predictors outperformed the null model in both experiments (likelihood‐ratio test: *χ* ^2^(3) = 65.4, *p* < 0.001 for the diurnal predator experiment and *χ* ^2^(3) = 42.7, *p* < 0.001 for the nocturnal predator experiment). However, model estimates indicated that birds exposed to a diurnal predator call did not show significant differences in food intake compared to the control group (Figure 2a). In contrast, birds exposed to a nocturnal predator call consumed significantly less food (estimates for treatment group: *β* = −1.48 g, 95% CI = −2.31 to −0.66, *p* < 0.001) compared to the control group (Figure 2b). Interestingly, the treatment group increased their daily food intake at a higher rate (effect of interaction between treatment group and day, *β* = 0.20 g/day, 95% CI = 0.14–0.26, *p* < 0.001) compared to the control group (Figure 2b). However, birds in the treatment group started with a lower food intake at the beginning of the experiment (8.2 g, SD = 0.3) compared to birds in the control group (9.5 g, SD = 0.3). By the end of the experiment, the treatment group's daily food intake increased to 10.8 g (SD = 0.3), while the control group maintained a constant food intake as the initial estimate (9.8 g, SD = 0.3). The treatment group reached the same level of food intake as the control group in the middle of the experiment (i.e. between days 8 and 9; Figure 2b).

Body condition models, which included group and interaction between group and day and group and food intake, explained significant variation in the response variables compared to a null model in both experimental settings (diurnal predator: *χ* ^2^(5) = 115.1, *p* < 0.001; nocturnal predator: *χ* ^2^(5) = 39.1, *p* < 0.001). However, significant differences between groups were only found in the model estimate for the nocturnal predator experiment, where the treatment group exhibited significantly lower body condition (*β* = −0.01, 95% CI = −0.03 to −1.39e‐03, *p* = 0.03) compared to the control birds (Figure 2d). For a bird with a wing length of 72.3 mm (the average length in our study), the model estimate for body condition translates to a 1 g lower body mass in the experimental versus control group. This reduction in body mass in the treatment group is predicted to result in a flight range that is 50 km shorter in the treatment group compared to the control.

**FIGURE 3:**
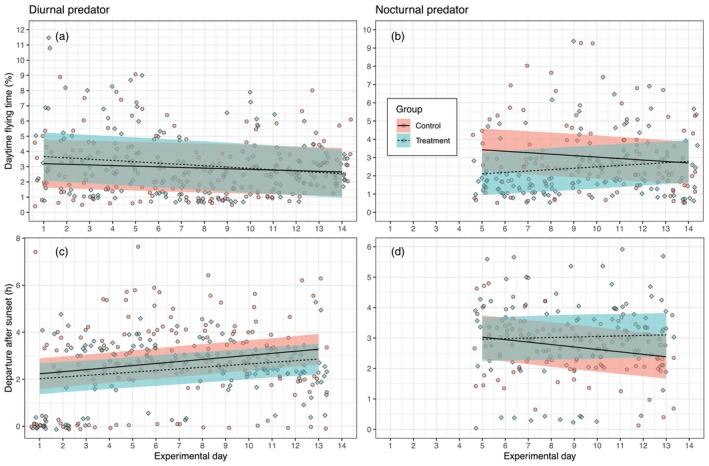
Changes in daily daytime activity (i.e. time spent in flight mode) and departure time relative to local sunset of first‐year migratory European robins (*Erithacus rubecula*) monitored in two experiments on separate groups of individuals under the playback calls of a non‐predator animal (control) and either a diurnal or nocturnal avian predator species (treatment). Black lines represent linear mixed‐effects model predictions, and shaded areas indicate the 95% confidence intervals of models that explained significant variation in the response variables compared to a null model. Individual raw data points are also displayed in the background. Models' construction, likelihood‐ratio test, and significant model estimates are reported in the text. In the second experiment (i.e. nocturnal predator), the first 4 days are missing due to a technical problem with the video‐recording equipment.

Activity levels, expressed as the percentage of time spent in flight mode every day, were better modelled when including both group and the interaction between group and day (i.e. changes over time) compared to a simpler null model for both experiments. This was true for 24 h activity (diurnal predator: *χ* ^2^(3) = 125.3, *p* < 0.001; nocturnal predator: *χ* ^2^(3) = 60.5, *p* < 0.001) and when separated for daytime (diurnal predator: *χ* ^2^(3) = 11.4, *p* < 0.01; nocturnal predator: *χ* ^2^(3) = 7.9, *p* < 0.05) and nighttime periods (diurnal predator: *χ* ^2^(3) = 184.0, *p* < 0.001; nocturnal predator: *χ* ^2^(3) = 50.1, *p* < 0.001). However, for the diurnal predator experiment, there were no significant differences between experimental and control groups (Figures S1 and S3; Figure 3a). In contrast, for the nocturnal predator experiment, a significant difference was found between groups for daytime activity (estimate for the treatment group, *β* = −2.09%, 95% CI = −3.73 to −0.46, *p* = 0.01; Figure 3b), but not when considering the entire 24 h activity period (Figure S2) or just the nighttime activity (Figure S4). The lower daytime activity observed in the treatment group for the nocturnal predator experiment correlates with reduced food intake (compare Figure 2b with Figure 3b), supporting our observation in the actogram (Figure 1) that foraging is expected to primarily occur during daytime.

Departure time was also better modelled when including group and interaction between group and day as predictors in both experiments (diurnal predator: *χ* ^2^(3) = 15.4, *p* = 0.001; nocturnal predator: *χ* ^2^(3) = 7.9, *p* < 0.05). However, no significant effects were found for the diurnal predator experiment (Figure 3c). In the nocturnal predator experiment, a significant interaction between group and day was found for the control group (*β* = −0.08, 95% CI = −0.14 to −0.02, *p* = 0.01). This negative slope indicates that control birds tended to leave 5 min earlier than the day before, resulting in a cumulative advance of about 40 min over the 9 days considered (Figure 3d). This pattern correlates with an increase in body condition over time (Figure 2d), suggesting that birds with higher body mass tend to depart earlier (Figure 3d). In contrast, the treatment group did not show a significant change in departure time over the course of the experiment relative to the model intercept, indicating that their departure times remained constant relative to the local sunset throughout the experiment, reaching 1 h later departure relative to the control at the end of the experimental period (Figure 3d).

When we modelled the duration of nocturnal restlessness, we could not find a model with fixed predictors, with and without interaction terms, that was better than the null intercept model. Hence, the early departure after sunset in the control group of the nocturnal predator had no effect on the duration of the nocturnal migratory activity (this analysis) nor on the overall nighttime activity (analysis above; Figure S4).

To determine whether the morning activity peak occurred before or after local sunrise, we identified for each bird and each day whether the activity level was higher or lower 1 h before or after sunrise, respectively. We then modelled the probability of the morning activity peak occurring after sunrise using a binomial distribution within a generalized mixed‐effects model. However, models for both experiments, including fixed effects, were never better than the null intercept model. The majority (~70%) of activity peaks occurred after the local sunrise, with no differences between experiments or experimental groups. Therefore, the peak activity observed in the actogram (Figure 1) at sunrise rather appears to be a response to the environmental light cue and it was not influenced by our experimental set‐up.

To determine if the settling time—the period when birds are active in the morning before entering their resting phase (as shown in Figure 1)—was part of a response strategy to the presence of predators, we estimated the settling time as the time when 50% of the cumulative daytime activity was reached. Models including fixed effects did not fit better than the null model, indicating no differences between the two experimental groups in both experiments. A sensitivity analysis using lower and higher cumulative activity thresholds for defining settling time yielded consistent results. Therefore, as for sunrise activity peak (previous paragraph), daytime settling time is strongly connected to the local sunrise time and does not appear to be influenced by stopover strategies related to our experimental conditions.

## 4 DISCUSSION

### 4.1 No response to the diurnal predator

According to our hypothesis, birds exposed to diurnal avian predator calls did not show significant differences in food intake compared to birds in the control group and did not adjust their daily activity pattern. Sparrowhawk attacks usually occur aloft, where manoeuvrability is less affected by body mass compared to take‐off performances from the ground or a perching position (Hedenström, 1992; Hedenström & Rosén, 2001). By maintaining an opportunistic foraging behaviour, with short bursts of activity when food is found and captured, robins can probably cope with the presence of a large avian predator during the daytime by relying on visual cues to assess and respond to imminent risks of attacks without compromising escaping ability (Walters et al., 2017). It is possible, in fact, that during the day, auditory cues signalling the presence of an avian predator are not ranked as highly important by songbirds. The soundscape in a forest during daylight hours is complex and varied, making it difficult for birds to precisely quantify the local presence of different potential predators. Additionally, daytime predator vigilance may also be shared among individuals, further diminishing the reliance on auditory cues as an anti‐predatory strategy (Elgar, 1989).

### 4.2 Food intake and body condition are affected by nocturnal predator

In contrast to their response to diurnal predators, robins exposed to nocturnal avian predator calls reduced their foraging activity, fuel intake and overall body condition relative to the control group. This significant reduction in food intake suggests that robins may decrease their foraging activity due to perceived predation risk. Indeed, the pressure from nocturnal predators, such as tawny owls (Mikkola, 1983), tested in this study, or the greater noctule bat (*Nyctalus lasiopterus*), which specializes in catching migratory songbirds at night, can be particularly high in certain habitats (Gong et al., 2021; Ibáñez et al., 2001). Furthermore, recent studies suggest that songbirds are even able to hear the low‐frequency audible portion of the bats' echolocation calls but do not show any escape response based on close‐range auditory cues alone (Gong et al., 2023). Hence, auditory cues may be used to alert to the presence of a predator approaching, but escaping responses are only occurring in the dark when the predator is at touching distance to its songbird prey (Gong et al., 2023). With such limited response time, escape reactions must be maximized by keeping fuel load to a minimum (Hedenström, 1992; Hedenström & Rosén, 2001; Lind et al., 2010; Walters et al., 2017).

A slower fuelling rate negatively affects migration speed (Alerstam & Lindström, 1990; Bayly, 2006; Lindström, 2003), potentially leading to later arrival at stopovers and wintering areas, which can carry over effects on the fitness of the individuals (Alerstam & Lindström, 1990; Lindström, 2003). The lower body condition in the nocturnal predator group highlights the potential negative impacts of predator presence on migratory birds' fitness and flight range. Reduced body mass can potentially affect stopover duration, flight range, migration success and overall survival.

We estimated a 50 km shorter flight range for the experimental group exposed to predator cues. However, this is likely an underestimation of the actual difference in fuel load. Our sample consisted of local birds captured in the stopover area, representing a random subset of the local population of birds available at this time of the season. Notably, the body condition at the beginning of Experiment 1 was higher relative to Experiment 2 (cf. Figure 2a,b). Future experimental procedures, we suggest, could be improved by capturing a larger number of birds and selecting only those with minimal fat load and body mass close to the lean body mass for the European robin (Pettersson & Hasselquist, 1985).

### 4.3 Delayed departure under predation risk at night

Contrary to our prediction, birds exposed to nocturnal predation risk delayed their departure time after sunset compared to the control group, rather than anticipating it as hypothesized. Control birds in the nocturnal predator experiment departed earlier each day, possibly due to improved body condition toward the end of the experiment, while the treatment group maintained constant departure times.

Robins may depart earlier to leave riskier predator territories and search for safer locations (Jenni & Schaub, 2003; Mueller et al., 2016), as demonstrated in blackcaps (*Sylvia atricapilla*; Fransson & Weber, 1997), albeit with reduced flight range potential. The control group's earlier departure times in the nocturnal predator experiment suggest a possible behavioural adaptation to increase stopover efficiency. On the other hand, the constant departure times in the treatment group indicate limited behavioural flexibility under predation stress.

We cannot exclude, however, that the experimental group did not anticipate their departure because of lower body condition. In other words, it is challenging to disentangle whether an earlier departure is a behavioural response to the presence of the predator (using the darker hours of the night for departure to reduce predator encounters) or whether birds are constrained by ending nocturnal activity at sunrise because landing and settling at the end of the migratory night must occur during daylight (see Section 4).

Nevertheless, the later departure will force birds to increase the number of stopovers, contributing to reduced overall migration speed (Alerstam & Lindström, 1990; Lindström, 2003). This reduction in migration speed can have significant carry‐over effects on fitness due to later arrival at subsequent stopovers and wintering territories (Alerstam & Lindström, 1990).

### 4.4 Correlation between increase of food intake and daytime activity

Under the perceived risk of the nocturnal predator, the daily activity levels and food intake were highly correlated, suggesting that food intake primarily occurred during the daytime and that the main activity during this period was foraging.

Initially, the food intake in the experimental group was significantly lower than that of the control group but increased significantly toward the end of the experiment. This change implies that the perceived risk was not a chronic stressor persisting throughout the experiment but rather an acute response to the presence of a predator upon arrival at an unknown stopover environment. The increase in food intake rate over time, on the other hand, is a potential compensatory mechanism following the acute response, resulting in greater food intake by the end of the experiment. The experimental group reached the same level of foraging activity and food intake as the control group approximately 1 week from the beginning of the experiment. This observation nicely aligns with the increased sensitivity to the danger of predation observed in black‐capped chickadee (*Poecile atricapillus*) 7 days after predator exposure (Zanette et al., 2019).

### 4.5 Consistency in nocturnal activity and sunrise‐related behaviours

We did not observe any differences in nocturnal activity duration or in activity related to sunrise times between experiments or experimental groups. Most morning activity peaks occurred after local sunrise, with no significant differences between the experimental groups. Similarly, settling time was closely linked to local sunrise and showed no differences between the groups, suggesting that these behaviours are not influenced by stopover strategies related to predator presence.

The lack of variation in settling time between experimental groups emphasizes the role of external environmental factors, such as light, in regulating daily activity patterns during migration, which, in our experimental setting, appears to override potential physiological or behavioural changes due to predator presence. This observation supports the notion that the timing of activity during migration is strictly regulated by both internal and external factors (Åkesson & Helm, 2020; Helm & Liedvogel, 2024), with limited flexibility, particularly in naïve first‐year migrants as tested in this study (Åkesson et al., 2021).

### 4.6 Future research directions

Nocturnal migratory robins live in the fear of predator attacks, and as experimentally revealed here, they may pay a cost in migratory performance for a safer migration to their wintering grounds (Alerstam, 2011; Alerstam & Lindström, 1990). Our study has also identified differences relative to the predator–diurnal versus nocturnal–and a relatively large variation in individual birds in their responses. There are gaps in the presented research that need to be filled. We have identified three potential areas for future study.

First, we need to develop a framework for investigating the long‐term effects of predation risk on migratory success and survival rates. This will provide deeper insights into the evolutionary pressures shaping migratory behaviour. Utilizing advanced tracking technologies to monitor real‐time movements and physiological states of migratory birds in response to predator presence could provide more detailed and accurate data. Furthermore, developing integrated models that incorporate behavioural, physiological and environmental data could improve predictions of migratory patterns and responses to changing predation landscapes.

Second, we need to explore additional methods to study the physiological and neurological mechanisms underlying the observed behavioural responses (e.g. Zanette et al., 2019; Zanette & Clinchy, 2020). It is unclear how the presence of a nocturnal predator can affect the behaviour and physiology of individuals during the daytime when no auditory calls are played. Stress hormone levels could potentially play a role in this dichotomy, or the activation of neuronal centres could lead to long‐lasting behavioural disorders (Zanette et al., 2019). Moreover, more detailed information could be obtained by measuring energy expenditure or other behavioural traits such as vigilance and the timing of foraging activities.

Third, conducting comparative studies could help determine the generalizability of these findings and identify species‐specific adaptations. The experimental setting used in this study should be extended to a larger number of migratory songbirds to understand how general behavioural and physiological responses are in predator‐induced fear for nocturnal and diurnal migrants. It is also important to investigate if similar responses occur during spring migration when competition for earlier arrival at breeding grounds is predicted to be even higher (Kokko, 1999). Moreover, exploring the role of other cues, such as chemical or visual signals, could provide a more comprehensive understanding of how migratory songbirds adapt to predation risk.

## 5 CONCLUSIONS

This study highlights the complex interplay between predation risk and migratory strategy in robins. Songbirds must balance various criteria during migration, including time, energy, and safety. Our research shows that the perceived risk of predation significantly influences these birds' physiology and behaviour without direct predator contact, but also that anti‐predatory strategies are differentially expressed in response to predators capture strategies.

The ecology of fear framework revealed here underscores the importance of understanding how predator presence shapes bird migration strategies and can impact individual fitness. This study contributes to a broader understanding of how migratory birds navigate and mitigate the various risks they encounter during their long migratory journeys.

## AUTHOR CONTRIBUTIONS

Giuseppe Bianco and Susanne Åkesson conceived the study; Xiaojia Wu performed the fieldwork with support from Sara Raj Pant, Susanne Åkesson and Giuseppe Bianco; Giuseppe Bianco extracted the activity data from videos; Giuseppe Bianco analysed the data with the help of Sara Raj Pant; Giuseppe Bianco, Sara Raj Pant and Susanne Åkesson wrote the manuscript.

## CONFLICT OF INTEREST STATEMENT

The authors have no competing interests.

## Supporting information


**Appendix S1:** Raw activity data extracted using computer vision for both experiments and each experimental house. Coloured lines represent activity scores for individual birds, identified by their unique ring numbers. The black line shows the mean activity for the house. The yellow shaded areas indicate daylight hours for each day.

**Figure S1:** Changes in daily activity levels (i.e., time spent in flight mode) of first‐year migratory European robins (*Erithacus rubecula*) under the playback calls of a non‐predator bird, the common crossbill (*Loxia curvirostra*; control) and the diurnal avian predator species Eurasian sparrowhawk (*Accipiter nisus*; treatment).
**Figure S2:** Changes in daily activity levels (i.e., time spent in flight mode) of first‐year migratory European robins (*Erithacus rubecula*) under the playback sound of a non‐predator animal, the deer (*Cervidae*; control) and the nocturnal avian predator species Tawny owl (*Strix aluco*; treatment).
**Figure S3:** Changes in daily nighttime activity levels (i.e., time spent in flight mode) of first‐year migratory European robins (*Erithacus rubecula*) under the playback calls of a non‐predator bird, the common crossbill (*Loxia curvirostra*; control) and the diurnal avian predator species Eurasian sparrowhawk (*Accipiter nisus*; treatment).
**Figure S4:** Changes in daily nighttime activity levels (i.e., time spent in flight mode) of first‐year migratory European robins (*Erithacus rubecula*) under the playback sound of a non‐predator animal, the deer (*Cervidae*; control) and the nocturnal avian predator species Tawny owl (*Strix aluco*; treatment).

## Data Availability

Data available from the Dryad Digital Repository https://doi.org/10.5061/dryad.k0p2ngfk6 (Bianco et al., 2025).
