# Supplementary material for: Nocturnal but not diurnal threats shape stopover strategy in a migrating songbird
Source: J Anim Ecol. 2025 May 23;94(7):1372–82. doi: 10.1111/1365-2656.70059 (PMC12214443; doi:10.1111/1365-2656.70059)
Supplement: Supplementary file 1 — Appendix S1: Raw activity data extracted using computer vision for both experiments and each experimental house. Coloured lines represent activity scores for individual birds, identified by their unique ring numbers. The black line shows the mean activity for the house. The yellow shaded areas indicate daylight hours for each day. [file JANE-94-1372-s002.pdf]

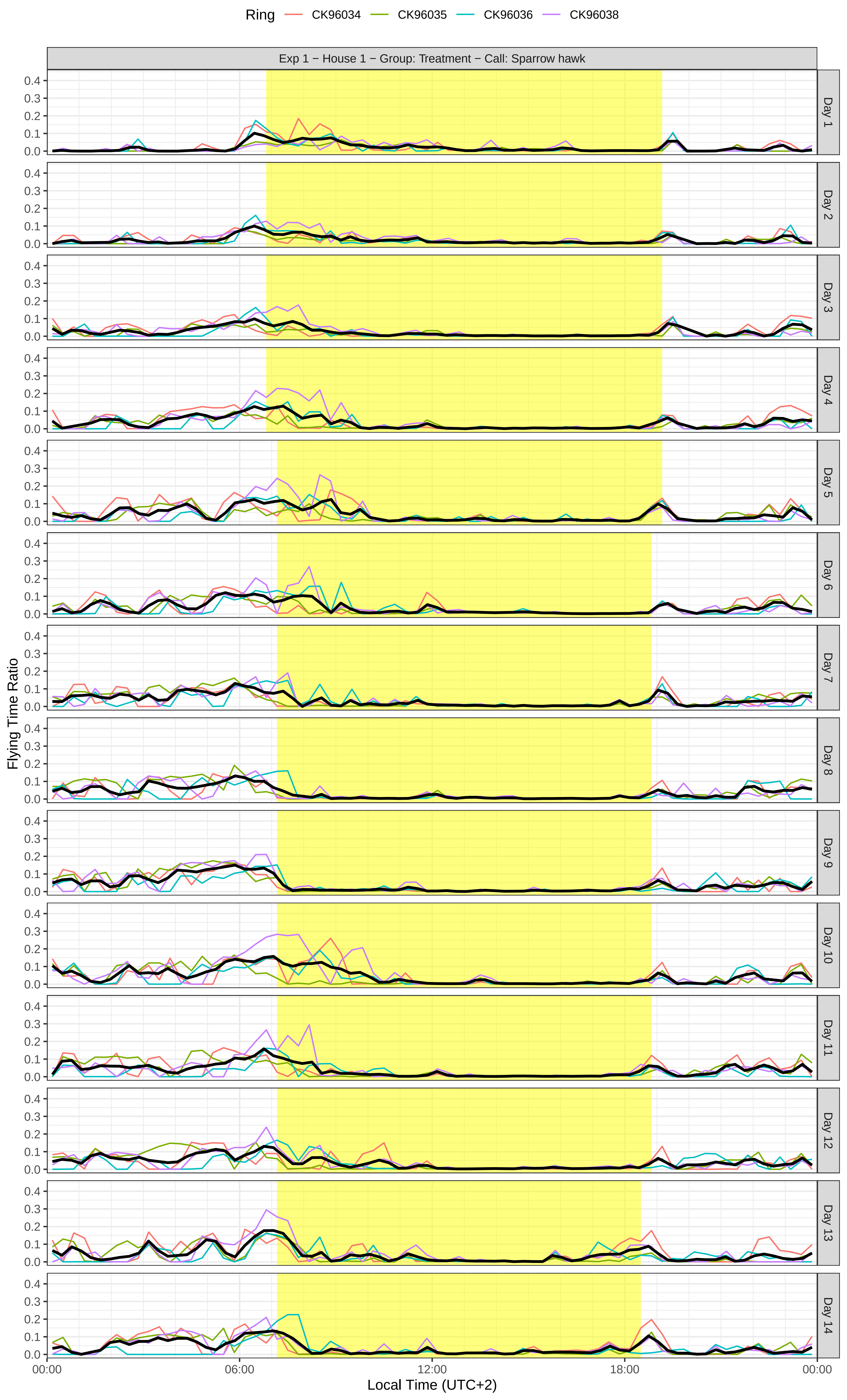

Ring
CK96034
CK96035
CK96036
CK96038
Exp 1 − House 1 − Group: Treatment − Call: Sparrow hawk
Day 1
Day 2
Day 3
Day 4
Day 5
Day 6
Day 7
Day 8
Day 9
Day 10
Day 11
Day 12
Day 13
Day 14
0.0
0.1
0.2
0.3
0.4
Flying Time Ratio
00:00
06:00
12:00
18:00
00:00
Local Time (UTC+2)

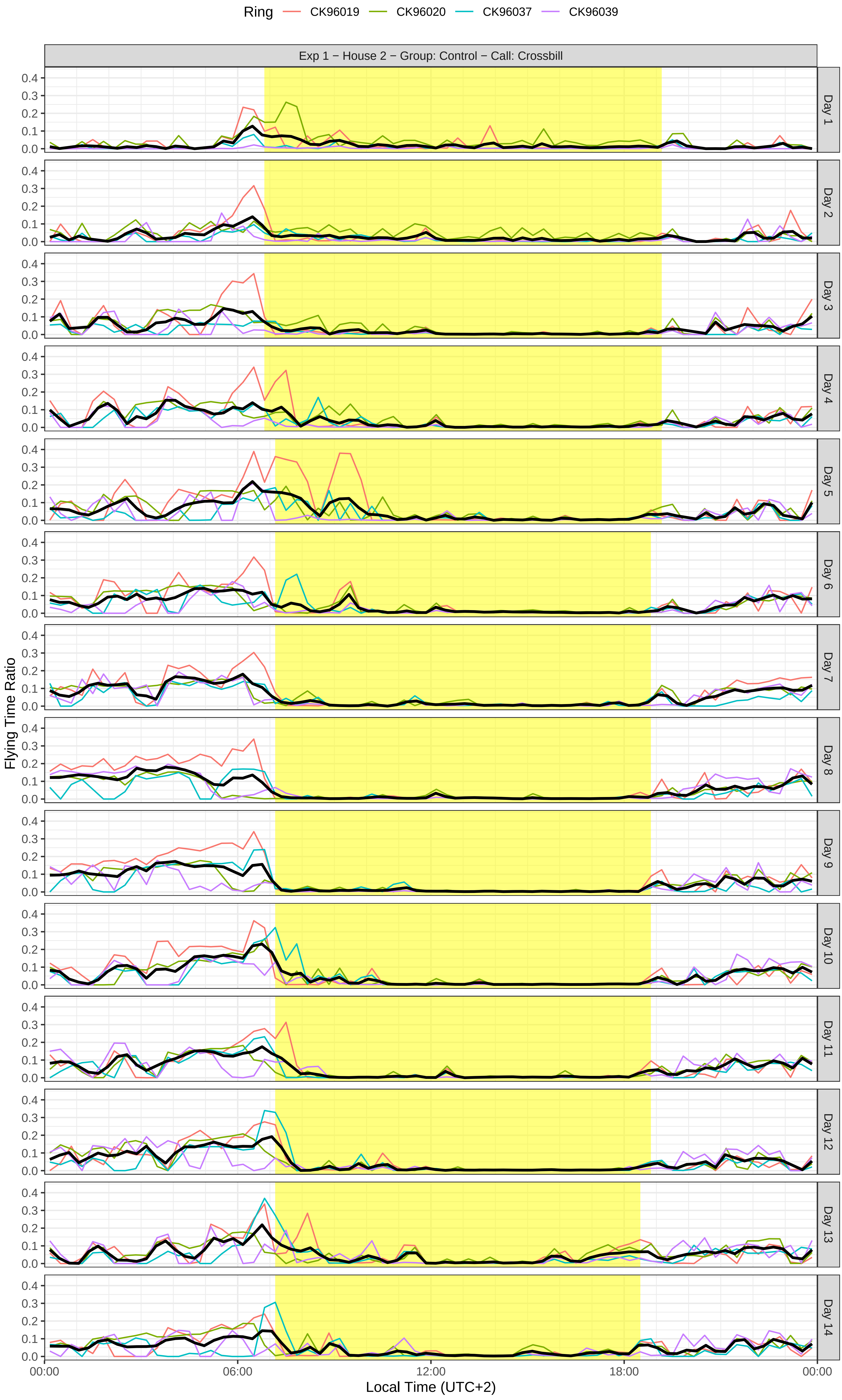

Ring
CK96019
CK96020
CK96037
CK96039
Exp 1 − House 2 − Group: Control − Call: Crossbill
Day 1
Day 2
Day 3
Day 4
Day 5
Day 6
Day 7
Day 8
Day 9
Day 10
Day 11
Day 12
Day 13
Day 14
0.4
0.3
0.2
0.1
0.0
Flying Time Ratio
00:00
06:00
12:00
18:00
00:00
Local Time (UTC+2)

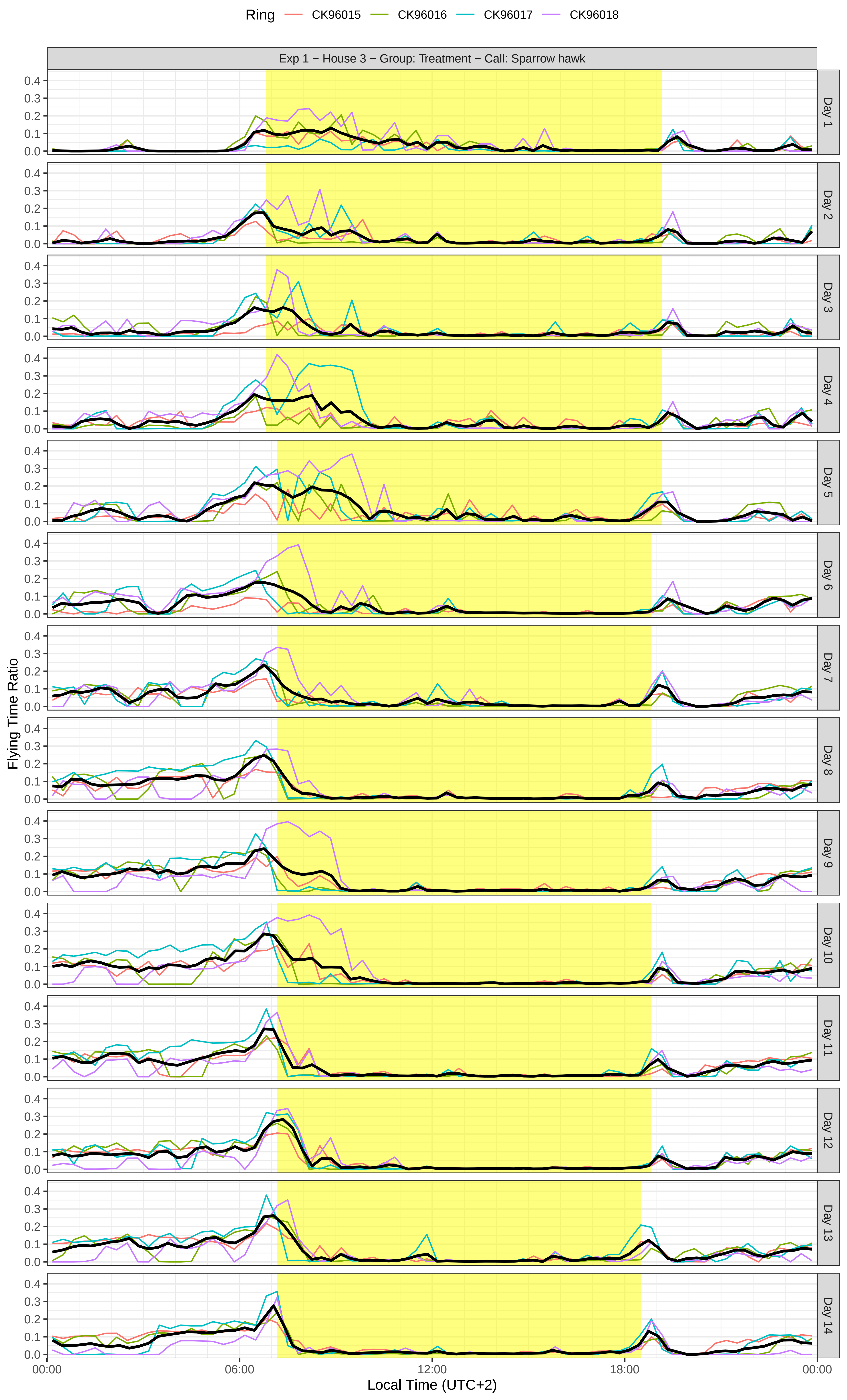

Ring
CK96015
CK96016
CK96017
CK96018
Exp 1 − House 3 − Group: Treatment − Call: Sparrow hawk
Day 1
Day 2
Day 3
Day 4
Day 5
Day 6
Day 7
Day 8
Day 9
Day 10
Day 11
Day 12
Day 13
Day 14
Flying Time Ratio
0.0
0.1
0.2
0.3
0.4
00:00
06:00
12:00
18:00
00:00
Local Time (UTC+2)

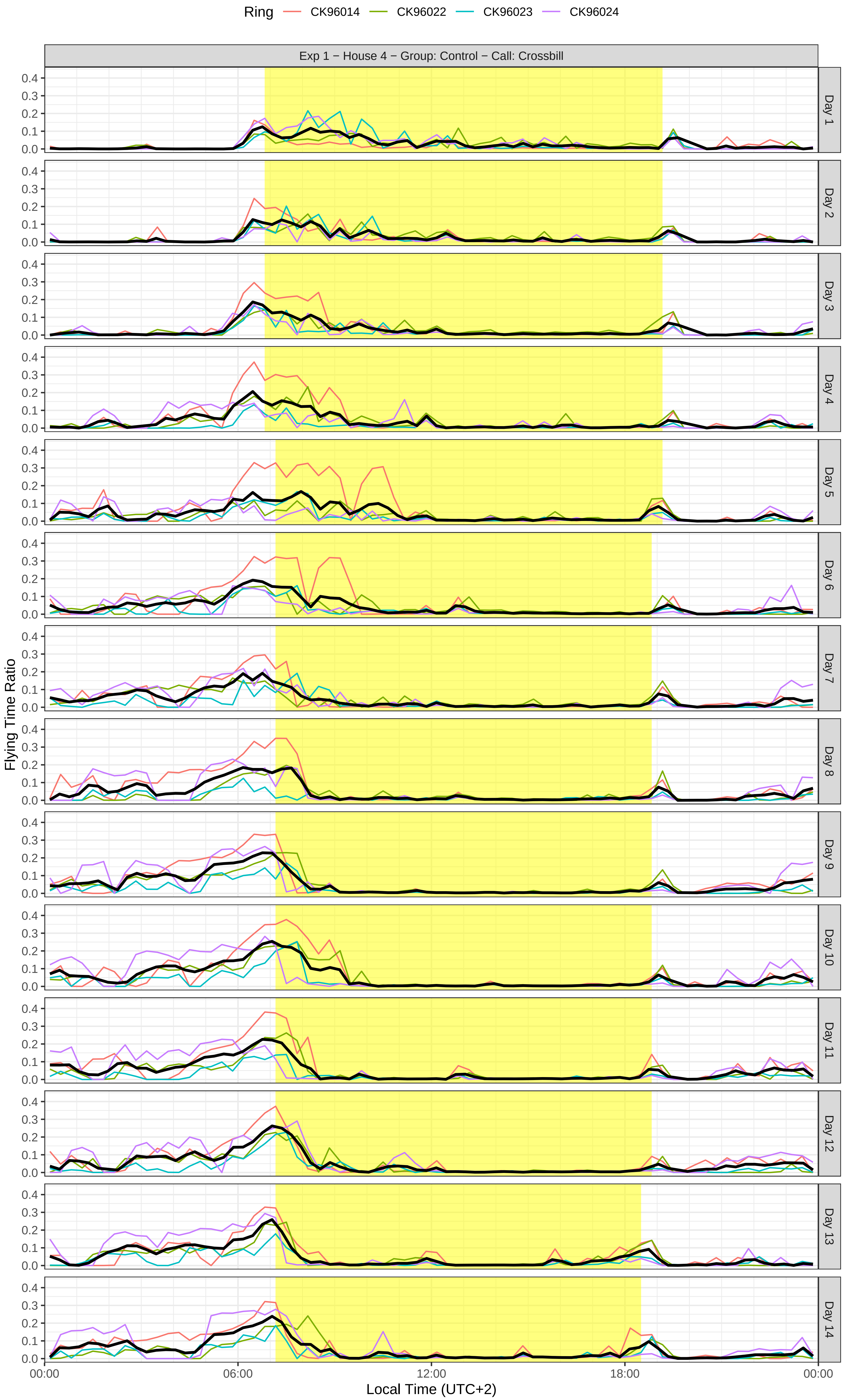

Ring
CK96014
CK96022
CK96023
CK96024
Exp 1 − House 4 − Group: Control − Call: Crossbill
Day 1
Day 2
Day 3
Day 4
Day 5
Day 6
Day 7
Day 8
Day 9
Day 10
Day 11
Day 12
Day 13
Day 14
Flying Time Ratio
0.0
0.1
0.2
0.3
0.4
00:00
06:00
12:00
18:00
00:00
Local Time (UTC+2)

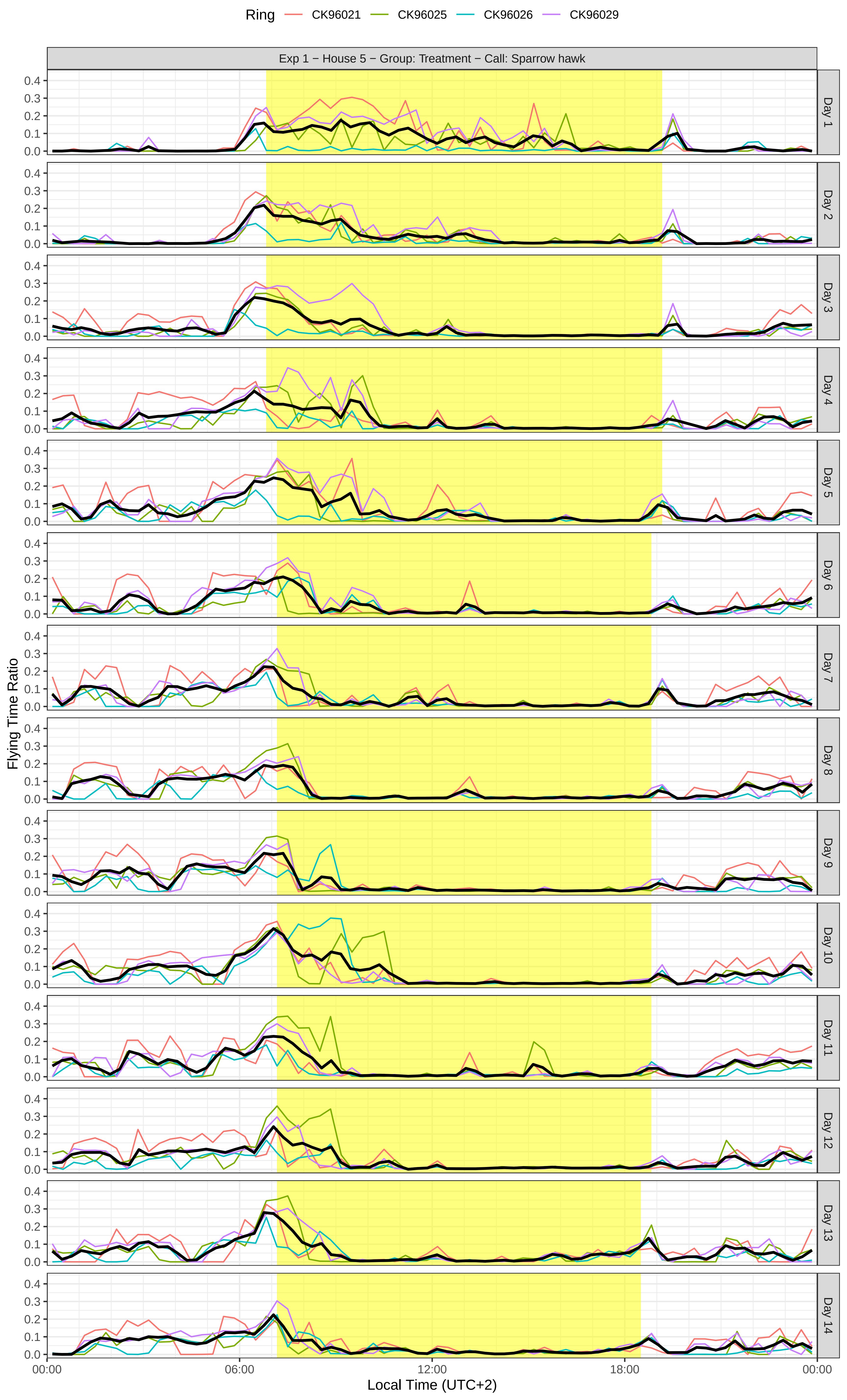
Ring
CK96021
CK96025
CK96026
CK96029
Exp 1 − House 5 − Group: Treatment − Call: Sparrow hawk
Day 1
Day 2
Day 3
Day 4
Day 5
Day 6
Day 7
Day 8
Day 9
Day 10
Day 11
Day 12
Day 13
Day 14
Flying Time Ratio
Local Time (UTC+2)
00:00
06:00
12:00
18:00
00:00

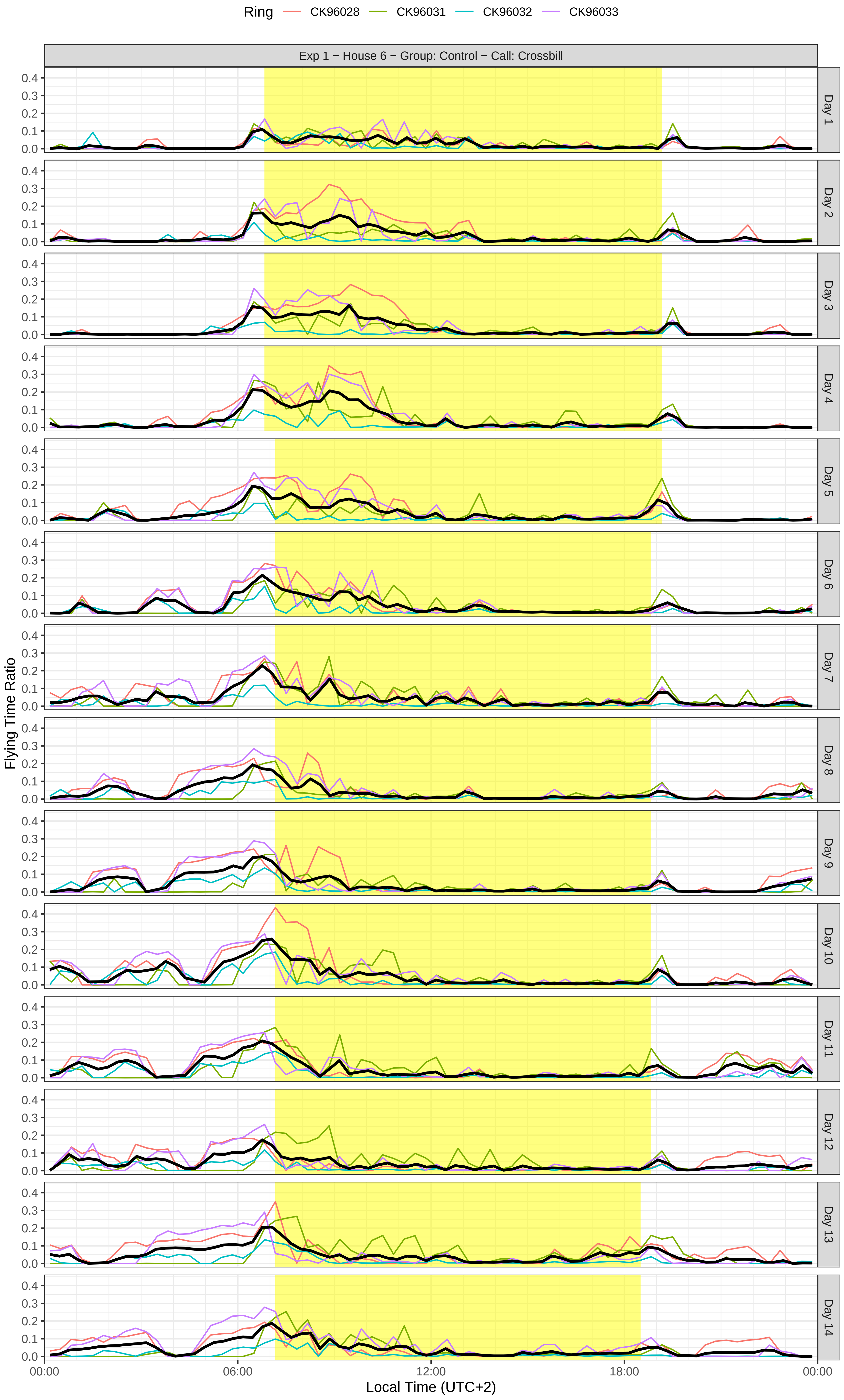

Ring
CK96028
CK96031
CK96032
CK96033
Exp 1 − House 6 − Group: Control − Call: Crossbill
Day 1
Day 2
Day 3
Day 4
Day 5
Day 6
Day 7
Day 8
Day 9
Day 10
Day 11
Day 12
Day 13
Day 14
Flying Time Ratio
0.0
0.1
0.2
0.3
0.4
00:00
06:00
12:00
18:00
00:00
Local Time (UTC+2)

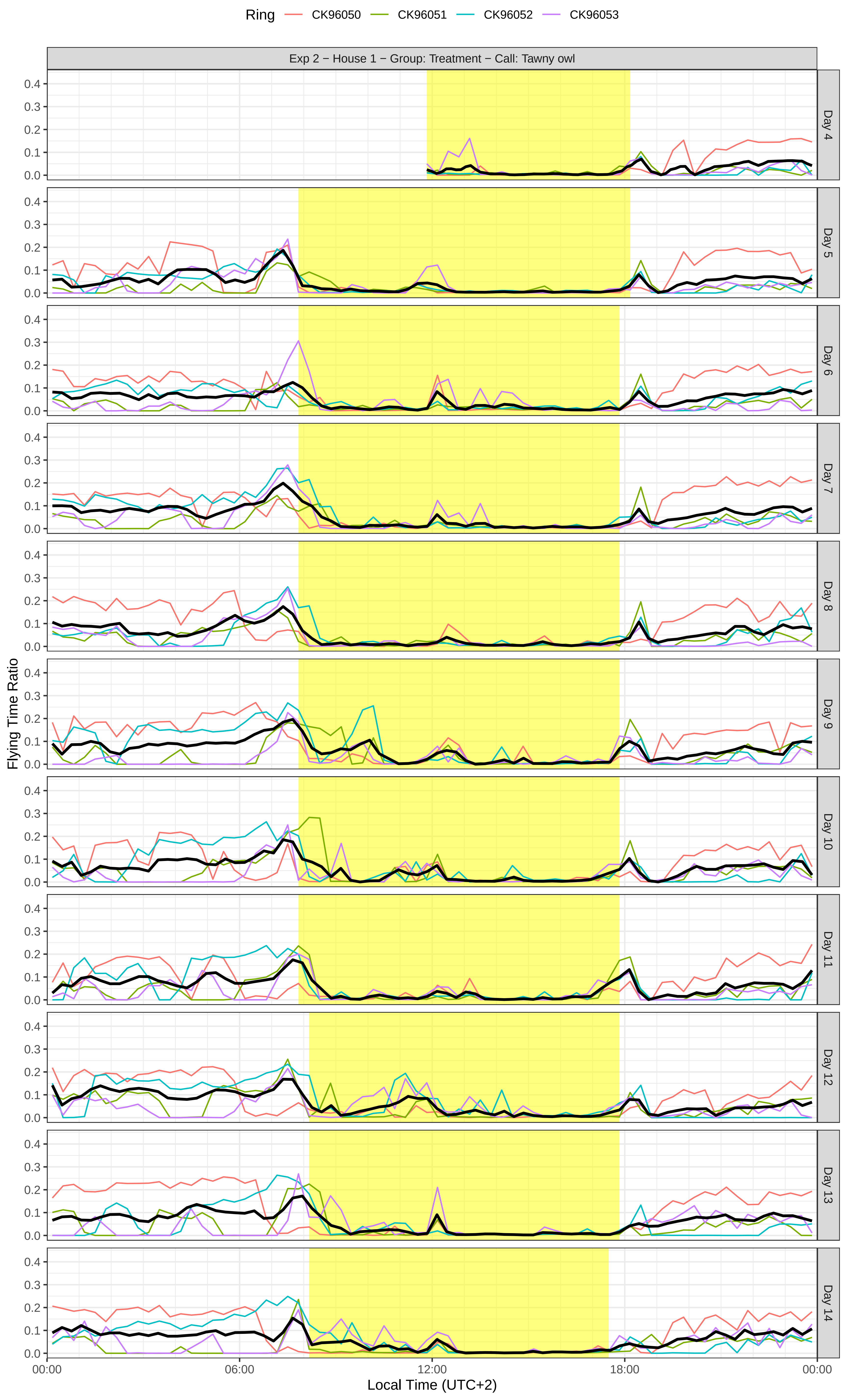

Ring
CK96050
CK96051
CK96052
CK96053
Exp 2 − House 1 − Group: Treatment − Call: Tawny owl
Day 4
Day 5
Day 6
Day 7
Day 8
Day 9
Day 10
Day 11
Day 12
Day 13
Day 14
0.0
0.1
0.2
0.3
0.4
Flying Time Ratio
00:00
06:00
12:00
18:00
00:00
Local Time (UTC+2)

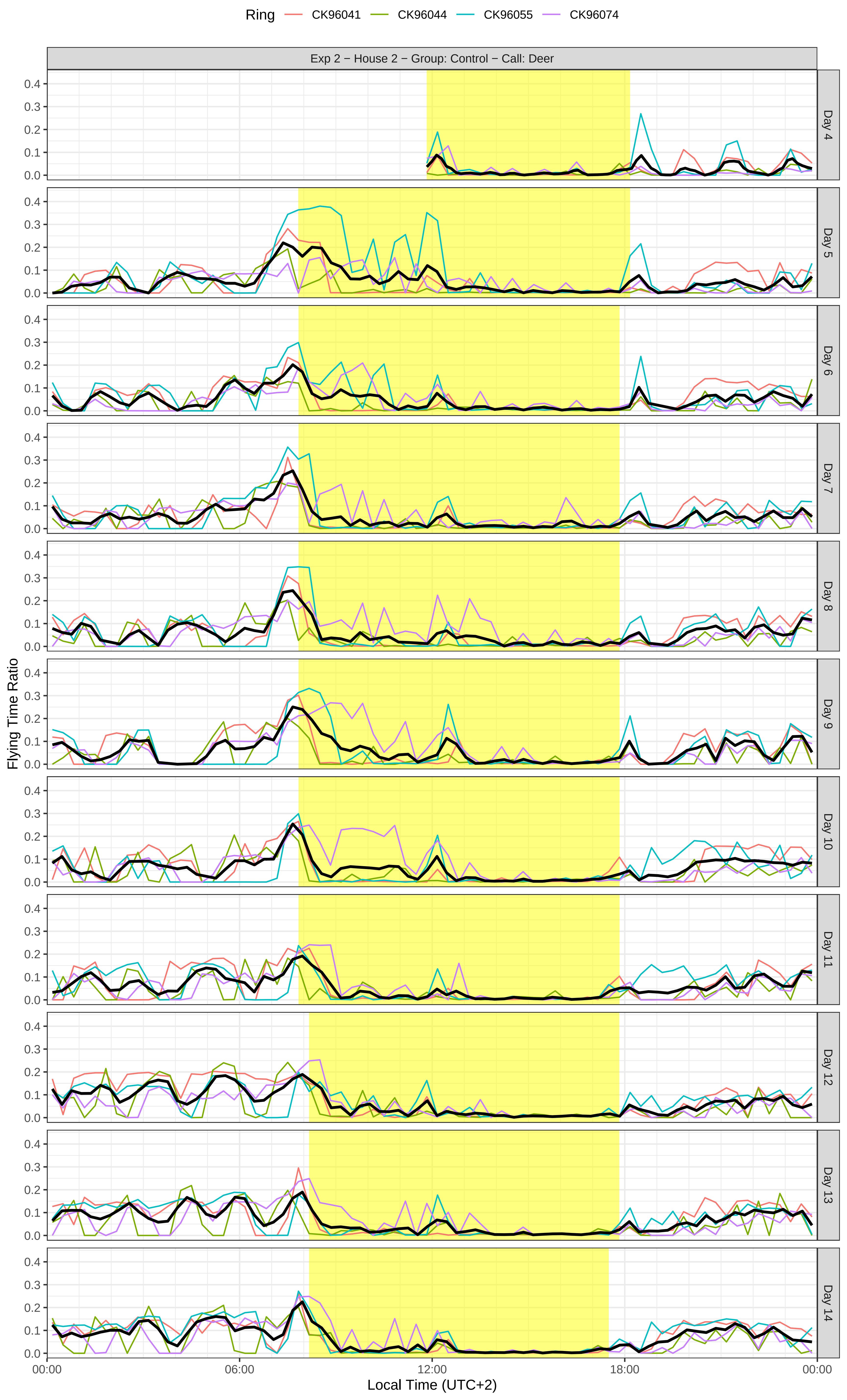

Ring
CK96041
CK96044
CK96055
CK96074
Exp 2 − House 2 − Group: Control − Call: Deer
Day 4
Day 5
Day 6
Day 7
Day 8
Day 9
Day 10
Day 11
Day 12
Day 13
Day 14
Flying Time Ratio
0.0
0.1
0.2
0.3
0.4
00:00
06:00
12:00
18:00
00:00
Local Time (UTC+2)

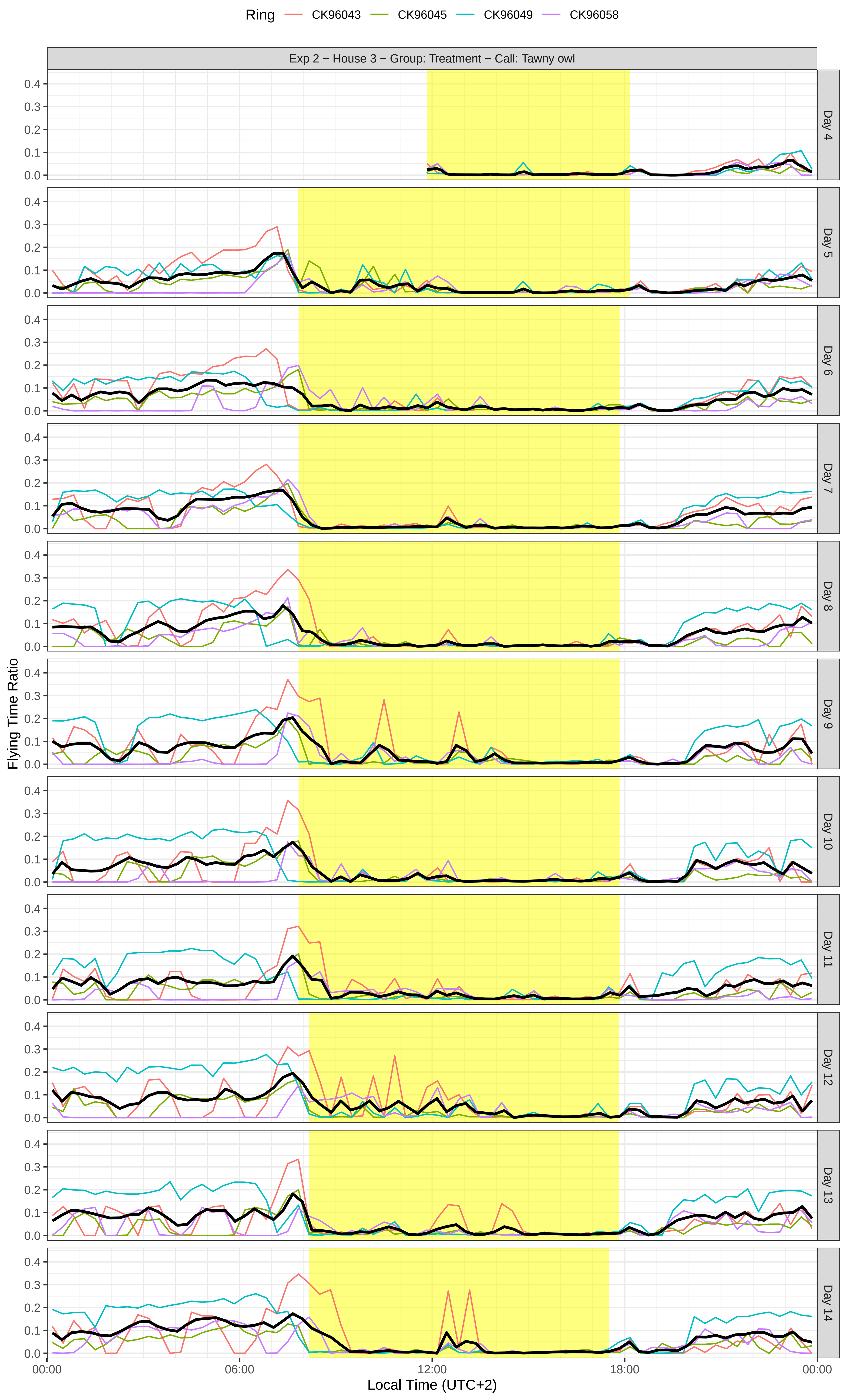

Ring
CK96043
CK96045
CK96049
CK96058
Exp 2 − House 3 − Group: Treatment − Call: Tawny owl
Day 4
Day 5
Day 6
Day 7
Day 8
Day 9
Day 10
Day 11
Day 12
Day 13
Day 14
Flying Time Ratio
0.0
0.1
0.2
0.3
0.4
00:00
06:00
12:00
18:00
00:00
Local Time (UTC+2)

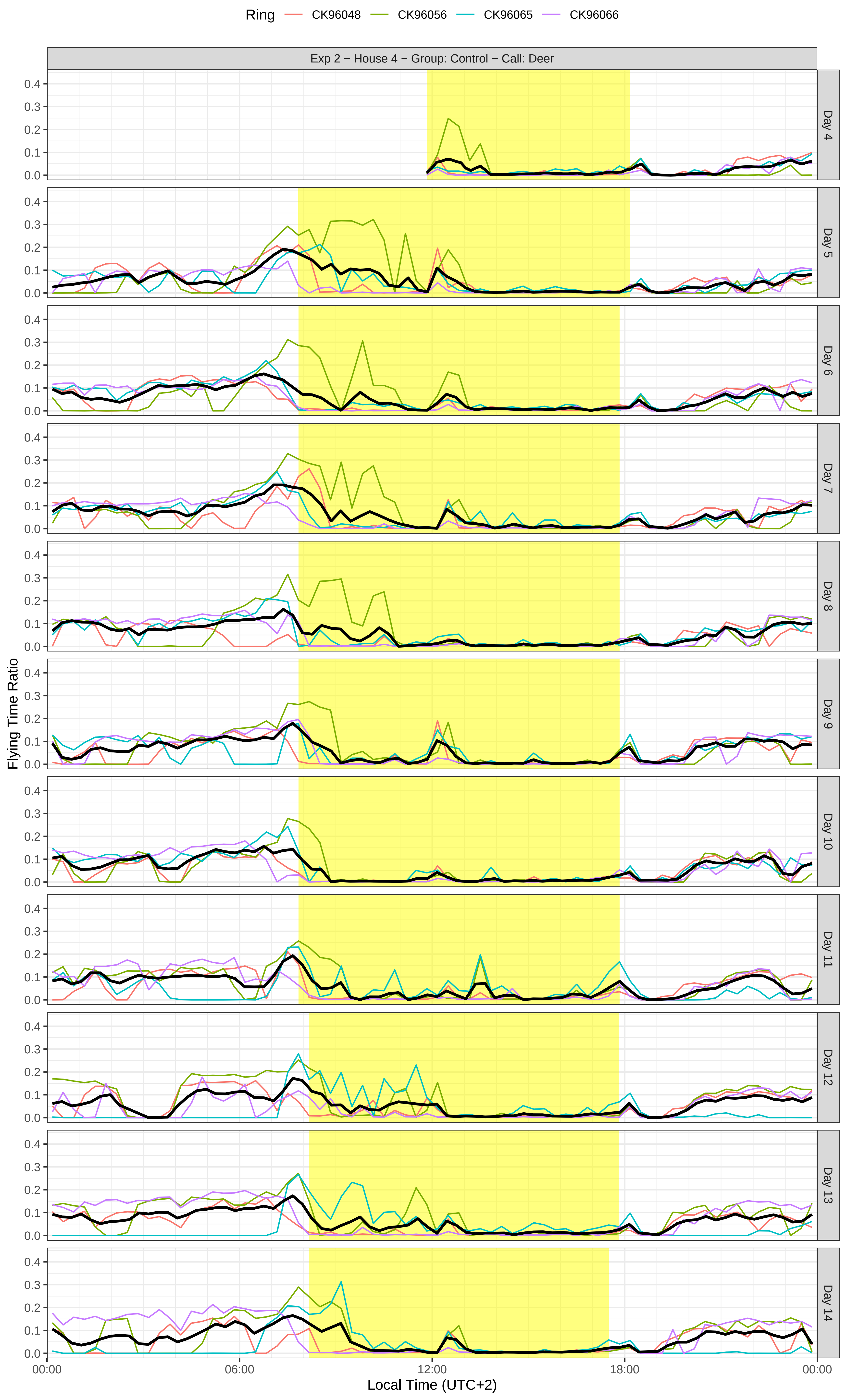

Ring
CK96048
CK96056
CK96065
CK96066
Exp 2 − House 4 − Group: Control − Call: Deer
Day 4
Day 5
Day 6
Day 7
Day 8
Day 9
Day 10
Day 11
Day 12
Day 13
Day 14
Flying Time Ratio
0.0
0.1
0.2
0.3
0.4
00:00
06:00
12:00
18:00
00:00
Local Time (UTC+2)

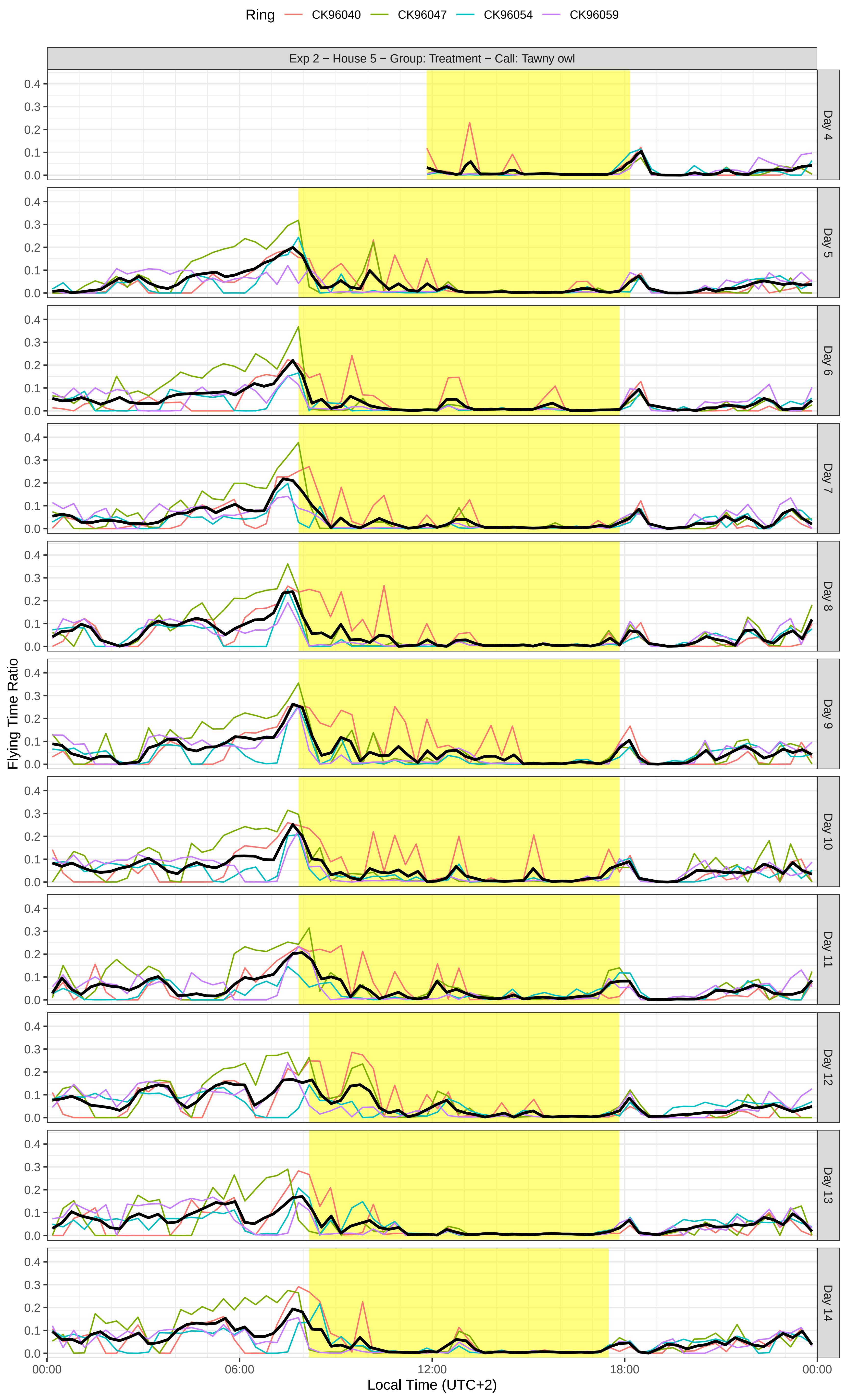
Ring
CK96040
CK96047
CK96054
CK96059
Exp 2 − House 5 − Group: Treatment − Call: Tawny owl
Day 4
Day 5
Day 6
Day 7
Day 8
Day 9
Day 10
Day 11
Day 12
Day 13
Day 14
Flying Time Ratio
0.0
0.1
0.2
0.3
0.4
00:00
06:00
12:00
18:00
00:00
Local Time (UTC+2)

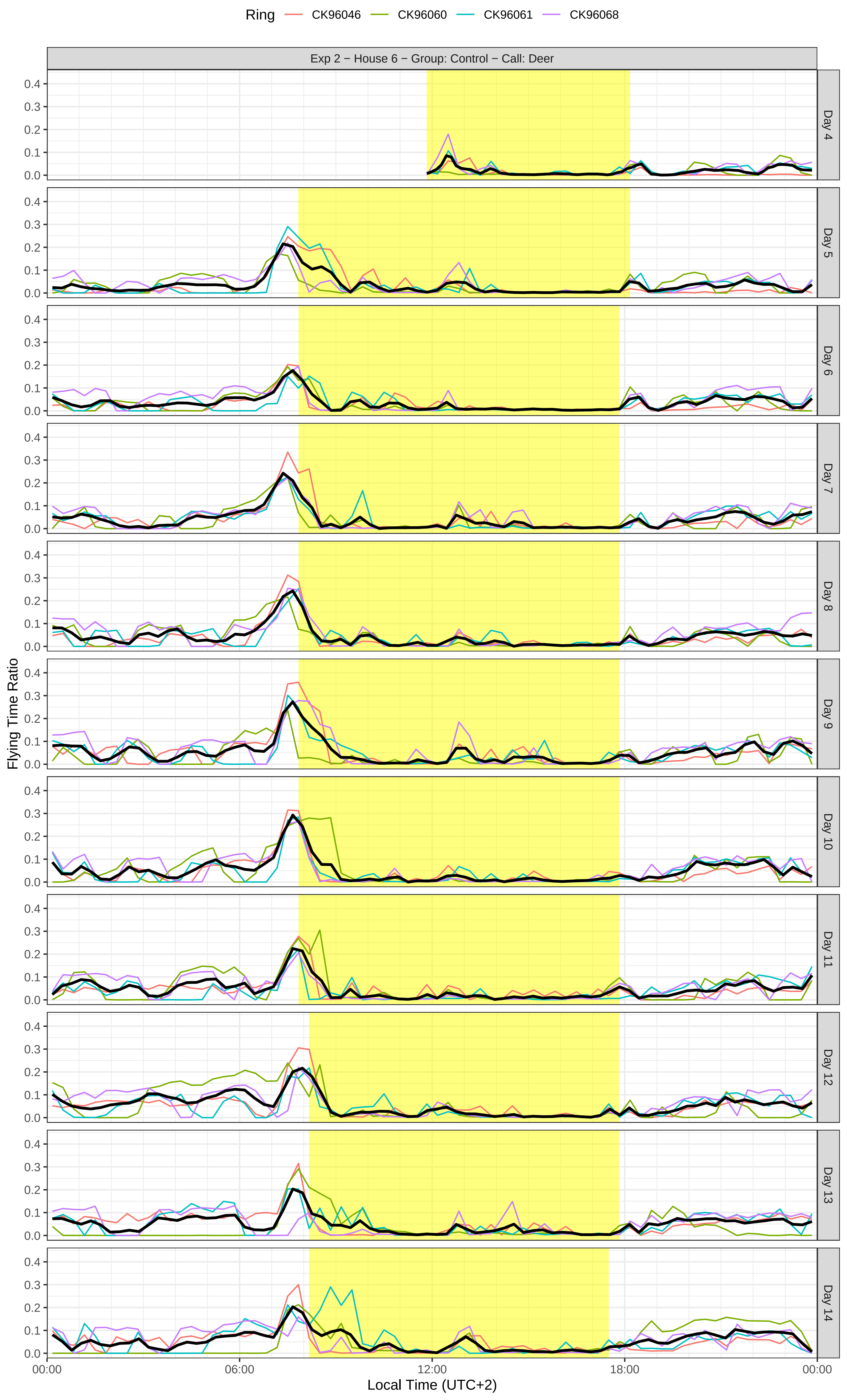

Ring
CK96046
CK96060
CK96061
CK96068
Exp 2 − House 6 − Group: Control − Call: Deer
Day 4
Day 5
Day 6
Day 7
Day 8
Day 9
Day 10
Day 11
Day 12
Day 13
Day 14
Flying Time Ratio
0.0
0.1
0.2
0.3
0.4
00:00
06:00
12:00
18:00
00:00
Local Time (UTC+2)
