# Supplementary material for: Nocturnal but not diurnal threats shape stopover strategy in a migrating songbird
Source: J Anim Ecol. 2025 May 23;94(7):1372–82. doi: 10.1111/1365-2656.70059 (PMC12214443; doi:10.1111/1365-2656.70059)
Supplement: Supplementary file 2 — Figure S1: Changes in daily activity levels (i.e., time spent in flight mode) of first‐year migratory European robins (Erithacus rubecula) under the playback calls of a non‐predator bird, the common crossbill (Loxia curvirostra; control) and the diurnal avian predator species Eurasian sparrowhawk (Accipiter nisus; treatment). Figure S2: Changes in daily activity levels (i.e., time spent in flight mode) of first‐year migratory European robins (Erithacus rubecula) under the playback sound of a non‐predator animal, the deer (Cervidae; control) and the nocturnal avian predator species Tawny owl (Strix aluco; treatment). Figure S3: Changes in daily nighttime activity levels (i.e., time spent in flight mode) of first‐year migratory European robins (Erithacus rubecula) under the playback calls of a non‐predator bird, the common crossbill (Loxia curvirostra; control) and the diurnal avian predator species Eurasian sparrowhawk (Accipiter nisus; treatment). Figure S4: Changes in daily nighttime activity levels (i.e., time spent in flight mode) of first‐year migratory European robins (Erithacus rubecula) under the playback sound of a non‐predator animal, the deer (Cervidae; control) and the nocturnal avian predator species Tawny owl (Strix aluco; treatment). [file JANE-94-1372-s001.pdf]

Supplementary Figures (S1—S4)

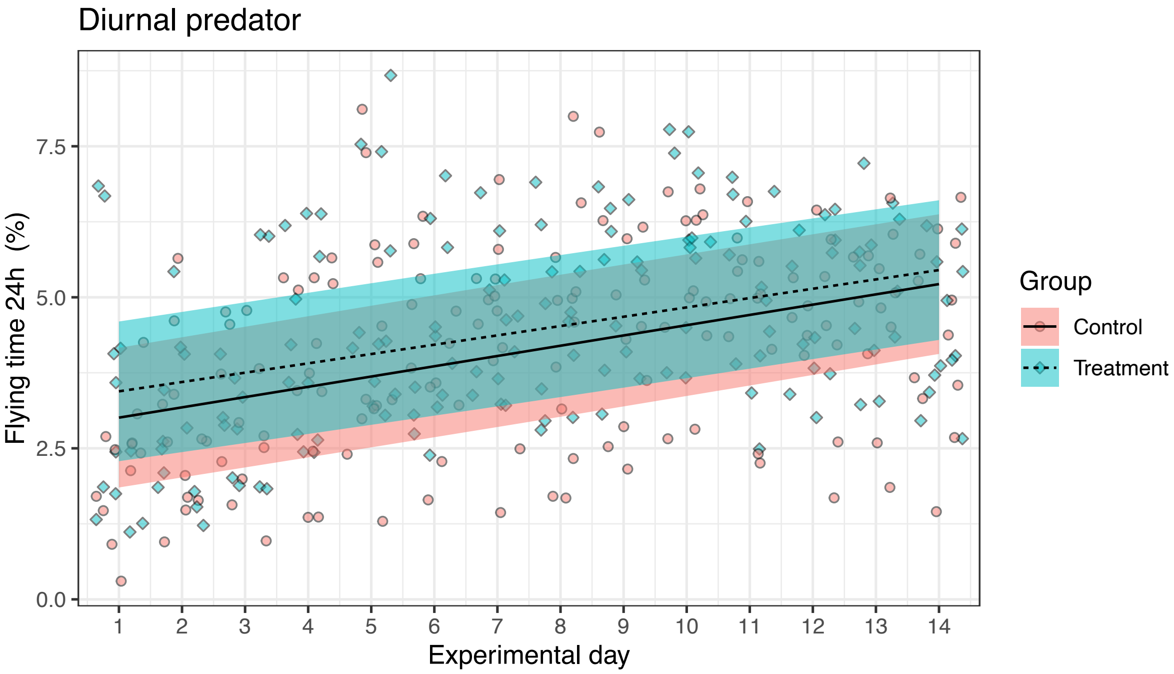

Figure S1. Changes in daily activity levels (i.e., time spent in flight mode) of first-year migratory European robins (*Erithacus rubecula*) under the playback calls of a non-predator bird, the common crossbill (*Loxia curvirostra*; control) and the diurnal avian predator species Eurasian sparrowhawk (*Accipiter nisus*; treatment). Black lines represent linear mixed-effects model predictions, and color-coded shaded areas indicate the 95% confidence intervals of models that explained significant variation in the response variables compared to a null model. Individual raw data points are also displayed in the background. Models' construction and likelihood-ratio test are detailed in the main text; however, model estimates were not significantly different between groups.

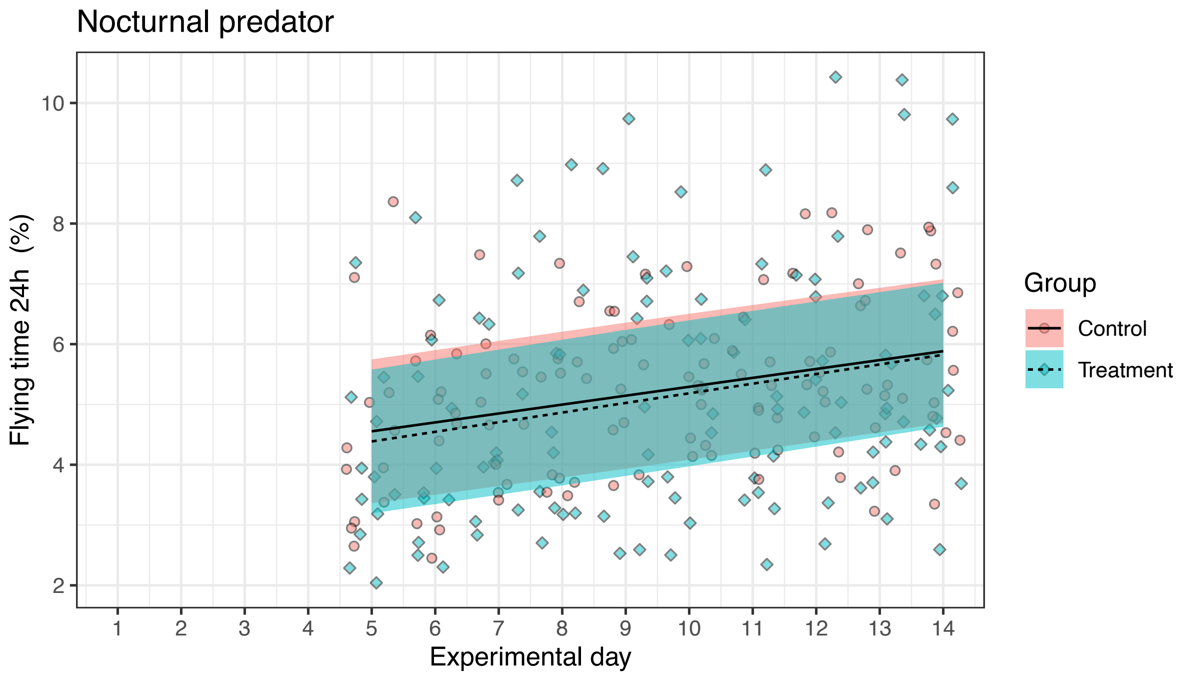

Figure S2. Changes in daily activity levels (i.e., time spent in flight mode) of first-year migratory European robins (*Erithacus rubecula*) under the playback sound of a non-predator animal, the deer (*Cervidae*; control) and the nocturnal avian predator species Tawny owl (*Strix aluco*; treatment). Black lines represent linear mixed-effects model predictions, and color-coded shaded areas indicate the 95% confidence intervals of models that explained significant variation in the response variables compared to a null model. Individual raw data points are also displayed in the background. Models' construction and likelihood-ratio test are detailed in the main text; however, model estimates were not significantly different between groups. The first 4 days are missing due to a technical problem with the video-recording equipment.

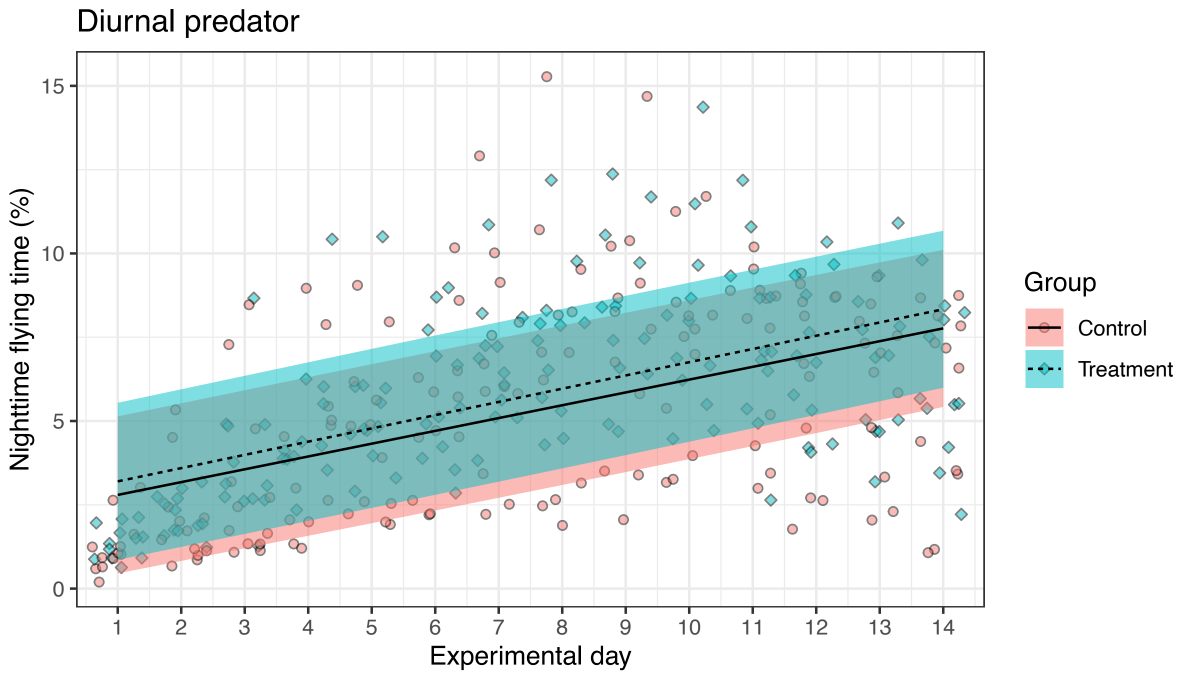

Figure S3. Changes in daily nighttime activity levels (i.e., time spent in flight mode) of first-year migratory European robins (*Erithacus rubecula*) under the playback calls of a non-predator bird, the common crossbill (*Loxia curvirostra*; control) and the diurnal avian predator species Eurasian sparrowhawk (*Accipiter nisus*; treatment). Black lines represent linear mixed-effects model predictions, and color-coded shaded areas indicate the 95% confidence intervals of models that explained significant variation in the response variables compared to a null model. Individual raw data points are also displayed in the background. Models' construction and likelihood-ratio test are detailed in the main text; however, model estimates were not significantly different between groups.

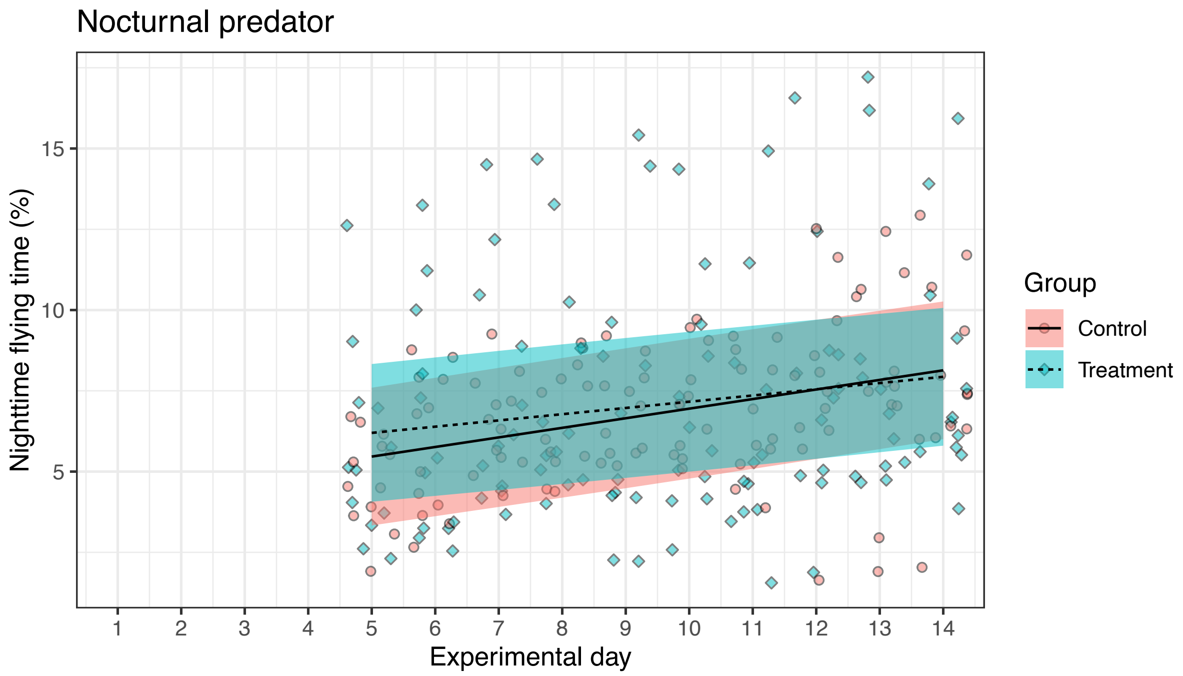

Figure S4. Changes in daily nighttime activity levels (i.e., time spent in flight mode) of first-year migratory European robins (*Erithacus rubecula*) under the playback sound of a non-predator animal, the deer (*Cervidae*; control) and the nocturnal avian predator species Tawny owl (*Strix aluco*; treatment). Black lines represent linear mixed-effects model predictions, and color-coded shaded areas indicate the 95% confidence intervals of models that explained significant variation in the response variables compared to a null model. Individual raw data points are also displayed in the background. Models' construction and likelihood-ratio test are detailed in the main text; however, model estimates were not significantly different between groups. The first 4 days are missing due to a technical problem with the video-recording equipment.
